# *BMPR2* Promoter Variants Effect Gene Expression in Pulmonary Arterial Hypertension Patients

**DOI:** 10.3390/genes11101168

**Published:** 2020-10-06

**Authors:** Jie Song, Katrin Hinderhofer, Lilian T. Kaufmann, Nicola Benjamin, Christine Fischer, Ekkehard Grünig, Christina A. Eichstaedt

**Affiliations:** 1 Laboratory for Molecular Genetic Diagnostics, Institute of Human Genetics, Heidelberg University, Im Neuenheimer Feld 366, 69120 Heidelberg, Germany; jie.song@csu.edu.cn (J.S.); katrin.hinderhofer@med.uni-heidelberg.de (K.H.); lilian.kaufmann@med.uni-heidelberg.de (L.T.K.); christine.fischer@med.uni-heidelberg.de (C.F.); 2 Department of Cardiovascular Medicine, The Second Xiangya Hospital of Central South University, Changsha 410011, China; 3 Centre for Pulmonary Hypertension, Thoraxklinik gGmbH Heidelberg at Heidelberg University Hospital, Röntgenstrasse 1, 69126 Heidelberg, Germany; nicola.benjamin@med.uni-heidelberg.de (N.B.); ekkehard.gruenig@med.uni-heidelberg.de (E.G.); 4 Translational Lung Research Centre Heidelberg (TLRC), German Centre for Lung Research (DZL), 69120 Heidelberg, Germany

**Keywords:** *BMPR2* promoter, pathogenic variant, heritable pulmonary arterial hypertension

## Abstract

Pathogenic variants have been identified in 85% of heritable pulmonary arterial hypertension (PAH) patients. These variants were mainly located in the bone morphogenetic protein receptor 2 (*BMPR2*) gene. However, the penetrance of *BMPR2* variants was reduced leading to a disease manifestation in only 30% of carriers. In these PAH patients, further modifiers such as additional pathogenic *BMPR2* promoter variants could contribute to disease manifestation. Therefore, the aim of this study was to identify *BMPR2* promoter variants in PAH patients and to analyze their transcriptional effect on gene expression and disease manifestation. *BMPR2* promoter variants were identified in PAH patients and cloned into plasmids. These were transfected into human pulmonary artery smooth muscle cells to determine their respective transcriptional activity. Nine different *BMPR2* promoter variants were identified in seven PAH families and three idiopathic PAH patients. Seven of the variants (c.-575A>T, c.-586dupT, c.-910C>T, c.-930_-928dupGGC, c.-933_-928dupGGCGGC, c.-930_-928delGGC and c.-1141C>T) led to a significantly decreased transcriptional activity. This study identified novel *BMPR2* promoter variants which may affect *BMPR2* gene expression in PAH patients. They could contribute to disease manifestations at least in some families. Further studies are needed to investigate the frequency of *BMPR2* promoter variants and their impact on penetrance and disease manifestation.

## 1. Introduction

Pulmonary arterial hypertension (PAH) is a rare disease characterized by remodeling of the small pulmonary vessels. This results in an increase of pulmonary artery pressure and resistance, eventually progressing to right heart failure [1]. In many forms of PAH, such as heritable (HPAH) or idiopathic PAH (IPAH), genetic defects have been identified [2]. In most cases of HPAH pathogenic variants (mutations) have been identified in the bone morphogenetic protein receptor 2 (*BMPR2*) gene leading to a loss of gene function [3]. The *BMPR2* gene encodes a cell membrane type II receptor of the transforming growth factor-β signaling pathway, which regulates expression of many target genes [4]. More than 600 pathogenic variants in the *BMPR2* gene have been identified so far, accounting for the disease development in about 85% of HPAH and 5–35% of IPAH patients [3]. These patients usually show an earlier onset of disease manifestation, more impaired hemodynamics [2] and reduced survival, compared to PAH patients without a genetic predisposition [5]. Apart from pathogenic variants in the *BMPR2* gene, genetic changes have also been described in recent years in the genes of its coreceptors *ACVRL1* and *ENG,* in the gene responsible for pulmonary venous occlusive disease *EIF2AK4*, and further BMPRII pathway genes [3]. So far, 17 genes have been classified as PAH-causing genes by the international task force for genetics and genomics in PAH [6].

While pathogenic variants in the *BMPR2* gene can lead to PAH, an incomplete penetrance of around 30% has been observed in family members with the same *BMPR2* variant [7]. In some families, up to 50% of *BMPR2* variant carriers can develop PAH [8]. It remains unclear why some variant carriers develop PAH while other carriers remain healthy for their entire life. It has been suggested that PAH patients could carry additional pathogenic variants in contrast to their healthy family members [9,10]. These so called “second hits” could serve as modifiers for disease penetrance leading to the disease manifestation together with the familial *BMPR2* variant [9]. Such second hits were also shown to be present in the noncoding or regulatory regions of the *BMPR2* gene [11]. However, in the normal routine genetic diagnostics setting, deep intronic regions and promoters are rarely investigated. Nevertheless, they can also contain pathogenic variants leading to PAH manifestation [12,13]. In previous studies, single substitution variants [14,15], a double substitution variant [13] and deleted or inserted tandem repeats [14,16] downstream of the transcription start site of the *BMPR2* gene were identified and led to reduced gene expression in a functional assessment. The contribution of *BMPR2* promoter variants to PAH manifestation, however, still remains unclear.

In this study we investigated whether *BMPR2* promoter variants in H/IPAH serve as second hits, by firstly identifying *BMPR2* promoter variants in H/IPAH patients and secondly, functionally characterizing the effect of the variants on *BMPR2* gene expression.

## 2. Materials and Methods

### 2.1. Study Population

PAH patients were clinically diagnosed by right heart catheterization following the guidelines of the European Society of Cardiology and the European Respiratory Society [1,17]. Further clinical examinations included medical history, physical examination, 12-lead electrocardiogram, chest radiograph, lung function testing and echocardiography at rest and during exercise. All patients of this study were either idiopathic or heritable PAH patients. In addition, healthy family members of H/IPAH patients performed echocardiography during exercise on a bicycle ergometer in the supine position. Pulmonary arterial systolic pressure (PASP) was measured with an increasing workload until maximum physical exhaustion was reached. A PASP value of >40 mmHg was used to define a hypertensive exercise response or a normal exercise response (PASP ≤ 40 mmHg) [18,19]. All subjects gave their informed consent for inclusion before they participated in the study. The study was conducted in accordance with the Declaration of Helsinki, and the protocol was approved by the Ethics Committee of the Medical Faculty of Heidelberg University (Project identification codes 065/2001 and S-426/2017).

### 2.2. Genetic Analysis

Genomic DNA was extracted from peripheral blood by an automated procedure (Autopure, Gentra Puregene Technology, Qiagen, Hilden, Germany). Variants in the *BMPR2* gene and the promoter region were identified either by direct Sanger sequencing (ABI 3130 genetic analyzer, ThermoFisher Scientific, Waltham, MA, USA) or by a PAH specific gene panel approach based on next-generation sequencing (Miseq, Illumina, San Diego, CA, USA) followed by Sanger sequencing confirmation [20]. Patients with no pathogenic variants in the *BMPR2* gene (RefSeq ID: NM_001204) were further evaluated by multiplex ligand-dependent probe amplification (MLPA) to search for large deletions or duplications (P093-C1, MRC-Holland, Amsterdam, the Netherlands). The PAH-specific gene panel of this study included 12 PAH genes (*ACVRL1*, *BMPR1B*, *BMPR2* including the *BMPR2* promoter region, *CAV1*, *EIF2AK4*, *ENG*, *KCNA5*, *KCNK3*, *SMAD1*, *SMAD4*, *SMAD9* and *TOPBP1*) and 17 further candidate genes as described previously [20]. Apart from coding exons, exon-intron boundaries reaching 20 bp into the introns were also analyzed. Each variant with a minor allele frequency in the database Ensembl < 1% was sought in the Human Gene Mutation Database and variants of *BMPR2, ENG, ACVRL1* and *SMAD4* also in the ARUP database. Putative transcription factor-binding sites containing promoter variants were assessed using the software MatInspector (version 3.9, Genomatix GmbH, München, Germany).

### 2.3. Plasmid Construction

Nine mutant 1520 base pair (bp) fragments plus the wild-type *BMPR2* promoter region (c.-1502 to c.18) were amplified from genomic DNA of PAH patients using a forward primer with a *Kpn*I restriction site at the 5′ end (5′-GAGGGTACCTCCCAAGCCATGCACATTTG-3′) and a reverse primer with a *Hind*III restriction site at the 3′ end (5′- CGCAAGCTTCTGCAGCGAGGAAGTCATC-3′) for cloning. The pGL4.10 (Promega GmbH, Walldorf, Germany) vector was cleaved by the *Kpn*I and *Hind*III restriction endonucleases and ligated with the ten amplified *BMPR2* promoter regions. The pGL4.10 vector included the firefly luciferase reporter gene sequence downstream of the inserted promoter region. The correct insertion of the recombinant gene constructs was confirmed by Sanger sequencing. The wild-type sequence contained no variants and corresponded to the human reference sequence (hg19). The pGL4.10 basic vector without any promoter element served as a negative control.

### 2.4. Cell Culture and Luciferase Assay

Commercial human pulmonary artery smooth muscle cells (PASMCs, Life Technologies GmbH, Darmstadt, Germany) were cultured in Medium 231 (Gibco, Thermo Fisher Scientific, Schwerte, Germany), supplemented with smooth muscle growth supplement (Gibco, Thermo Fisher Scientific, Schwerte, Germany) in a 37 °C, 5% carbon dioxide and 95% humidified cell culture incubator. Cells of passage 3 to 6 were plated onto 24-well plates at 5000 cells per well. After 24 h of incubation pGL4.10-*BMPR2*-variant constructs or pGL4.10-*BMPR2*-wild-type plasmids were added to the cells together with a transfection reagent (Lipofectamine 3000, Thermo Fisher Scientific, Schwerte, Germany). After 48 h of further incubation cells were lysed. Cleared cell lysates were aliquoted into 96-well plates. The activities of the firefly luciferase and the internal standard renilla luciferase were assayed with a Dual-Luciferase Reporter assay kit (Promega GmbH, Walldorf, Germany) using the auto-injector system Lucy2 Microplate Luminometer (Anthos Mikrosysteme GmbH, Krefeld, Germany). Each transfection was carried out five times and measured in triplicate by luciferase assay.

### 2.5. Statistical Analysis

Data were shown as mean ± standard deviation. Analysis of variance was performed by SPSS (version 21.0.0, IBM, Amonk, NY, USA), considering a *p*-value < 0.05 as statistically significant. Gene expression was normalized to the wild-type (corresponding to 1.0). Relative gene expression was calculated by: wild-type = value of every mutant plasmid/value of wild-type plasmid.

## 3. Results

### 3.1. Clinical Characteristics

Seven HPAH families with 53 family members and three IPAH patients were included in this study. The clinical characteristics of seven index patients were summarized in Table 1. The mean age of HPAH index patients was 34 years, 43% were female. The hemodynamic parameters measured by right heart catheterization revealed a mean pulmonary arterial pressure of 60.3 ± 9.4 mmHg, a pulmonary vascular resistance of 20.2 ± 5.7 Wood Units and a reduced cardiac index of 2.1 ± 0.9 mL/min/m^2^. Of 53 sequenced family members, PASP during exercise was measured in 39 individuals.

### 3.2. Genetic Analysis

**Figure 1:**
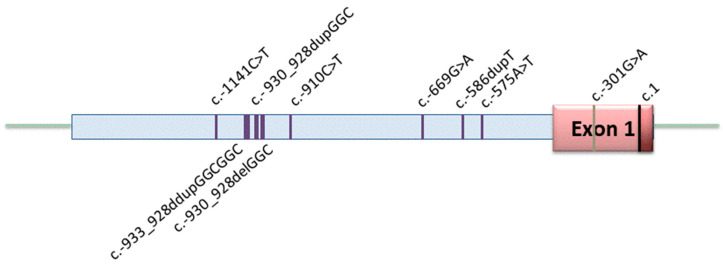
Distribution of variants in the promoter region of *BMPR2*. Nine variants were identified including five heterozygous substitutions, three heterozygous duplications/deletions at a single site, and one homozygous substitution (c.-586dupT). The 5′ untranslated region starts left of the nucleotide position c.1.

To detect promoter variants that may have an impact on gene expression and disease penetrance, a segment of 1520 bp of the 5′ untranslated region (UTR) upstream region of the *BMPR2* gene was analyzed. All subjects were included regardless of whether they carried any other pathogenic variant or variant of uncertain significance (VUS) in the coding area of *BMPR2* or any other analyzed PAH gene. In total, nine variants were discovered in the promoter region (Figure 1) of which four had not been described before in PAH patients (Table 2). Four heterozygous nucleotide substitutions (c.-301G>A, c.-575A>T, c.-910C>T and c.-1141C>T) and one heterozygous duplication (c.-933_-928dupGGCGGC) were detected in four HPAH families (see Table 2). Additionally, the previously described c.-669G>A variant was identified in three HPAH families. Among these three families, only two index patients carried the c.-669G>A variant, while in the third family, this variant was only present in healthy family members.

In two further IPAH patients, a duplication or a deletion of a three base pair repeat out of a twelve repeat GGC region was identified (c.-930_-928dupGGC and c.-930_-928delGGC). This resulted in 11 and 13 GGC repeats in contrast to 12 GGC repeats in the wild-type sequence. The last variant in the *BMPR2* promoter region was a homozygous duplication (c.-586dupT) present in a third IPAH patient. The locations of the nine identified variants are depicted in Figure 1.

### 3.3. Effect of Promoter Variants on Gene Expression

**Figure 2:**
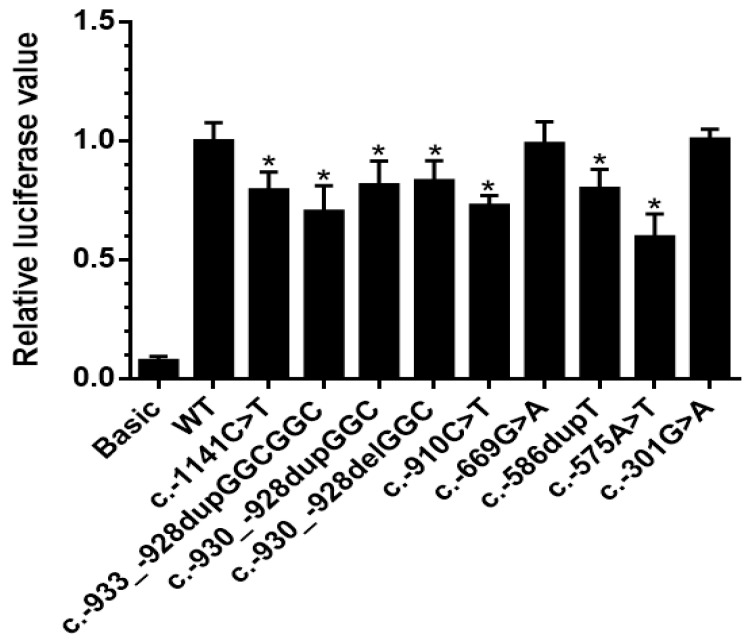
Transcriptional activity analysis of *BMPR2* promoter variants. The transcriptional activity of nine *BMPR2* promoter variants was compared with the wild-type *BMPR2* promoter by dual-luciferase assay. Seven of the nine variants showed significantly reduced gene expression in comparison to the wild-type. Basic: pGL4.10 plasmid (without promoter, negative control), WT: wild-type, c.-930_-928delGGC: 11GGC repeats, c.-930_-928dupGGC: 13GGC repeats, c.-933_-928dupGGCGGC: 14GGC repeats. Data are presented as mean ± standard error of the mean, normalized to the wild-type; *p*-value refers to the comparison between each mutant to wild-type; *: *p* < 0.05.

The effect of the nine different *BMPR2* promoter variants on the transcriptional activity of *BMPR2* was analyzed using a luciferase reporter gene assay after transient transfection of human PASMCs. The transcriptional activity of the variants was compared to the *BMPR2* wild-type promoter. The results revealed that apart from the two variants c.-669G>A and c.-301G>A, all plasmids with *BMPR2* promoter variants led to a statistically significant decrease of transcriptional activity compared to the plasmid carrying *BMPR2* wild-type promoter (Figure 2).

### 3.4. Association of Promoter Variants with an Abnormal Pulmonary Artery Pressure during Exercise or PAH Manifestation

**Figure 3:**
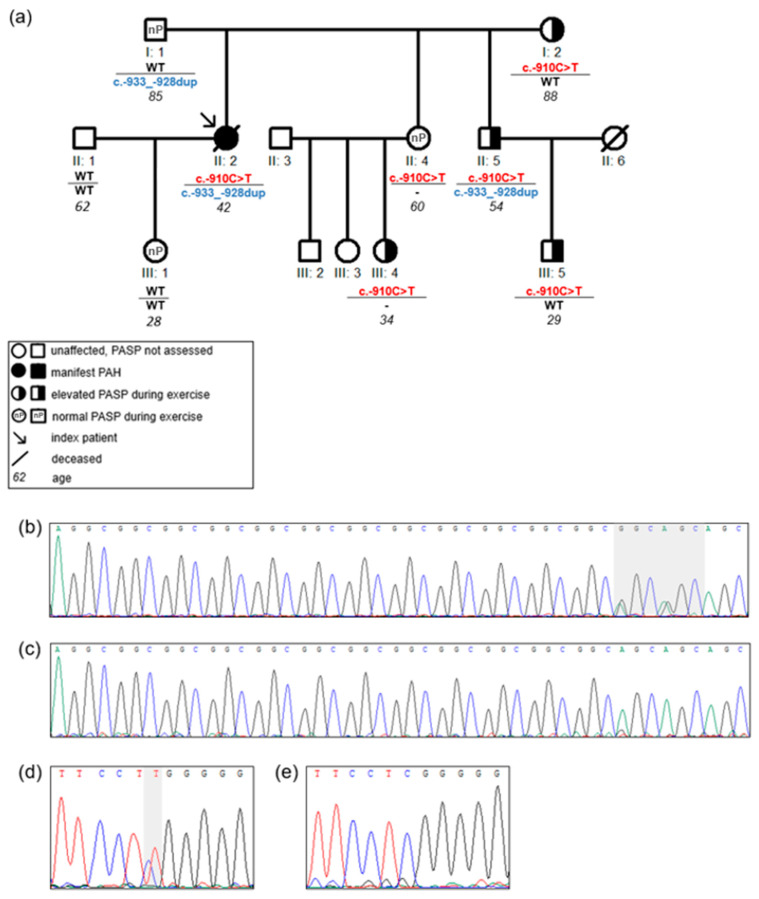
Pedigree of Family 1 with c.-910C>T and c.-933_-928dupGGCGGC in the 5′UTR of *BMPR2* gene: (**a**) the pedigree with *BMPR2* 5′UTR variants c.-910C>T and c.-933_-928dupGGCGGC. Only the c.-910C>T variant is present in all family members with an elevated pulmonary arterial systolic pressure (PASP) during exercise. The horizontal line separates the two loci in *BMPR2* 5′UTR c.-910C>T and c.-933_-928dupGGCGGC variants; c.-910C>T: *BMPR2* c.-910C>T; c.-933_-928dup: *BMPR2* c.-933_-928dupGGCGGC; WT: wild-type; -: not sequenced. Italic numbers represent the age of individuals; (**b**) sequencing analysis of the c.-933_-928dupGGCGGC shows the heterozygous G>A changes on the right side within the grey shaded area indicating a duplication of a 3 bp repeat; (**c**) c.-933_-928 wild-type sequence; (**d**) sequencing analysis of the heterozygous c.-910C>T variant within the grey shaded area; (**e**) c.-910 wild-type sequence.

One family (Family 1) was identified with two promoter variants in the index patient (Figure 3). The index patient (II: 2) was a 42-year-old woman, who was diagnosed with PAH 6 years prior to study inclusion. At diagnosis she presented with exertional dyspnea and limited exercise capacity. The echocardiogram revealed an enlarged right ventricle and right atrium. Right heart catheterization showed a mean pulmonary arterial pressure of 67 mmHg, pulmonary arterial wedge pressure of 5 mmHg, and pulmonary vascular resistance of 26 Wood Units. The patient carried the two variants c.-910C>T and c.-933_-928dupGGCGGC in the *BMPR2* promoter region. Each variant was inherited from one parent, thus being biallelic and compound heterozygous in the index patient (Figure 3). Both variants were shown to lead to a reduced gene expression in the functional analysis (Figure 2). Apart from the index patient, her brother (II: 5) also carried both promoter variants but did not develop PAH. The c.-910C>T variant was also found in her mother (I: 2) and in four other family members (II: 4, II: 5, III: 4, III: 5) without manifest PAH. In four of the five family members with the c.-910C>T variant an elevated PASP during exercise was measured by echocardiography, while only one variant carrier showed a normal pressure response (Figure 3, Family 1 in Table 3). This observation fits to the hypothesis of cosegregation of the variant with an elevated PASP assuming an autosomal dominant model of inheritance with reduced penetrance.

In contrast, the second *BMPR2* promoter variant (c.-933_-928dupGGCGGC) showed no cosegregation with abnormal PASP (Figure 3). Thus, while the first variant may be associated with a hypertensive exercise response the second variant does not seem to contribute to disease manifestation.

In the other HPAH families no cosegregation of the respective promoter variant with an abnormal PASP was apparent (Table 3). In one family (Family 3, Table 3) the promoter variant (c.-575A>T) was not present in the second HPAH patient of the family. In another family (Family 2, Table 3) a single, different promoter variant was identified in the two HPAH patients of the family. However, one of the two variants (c.-301G>A) revealed no effect on gene expression in the functional analysis. Promoter variants, which reduced gene expression were identified in six of the ten I/HPAH index patients (Table 3). Out of these six patients only three patients had a known pathogenic variant in the coding region of a PAH gene. Thus, in these patients the promoter variant could be considered a possible second hit adding to a known pathogenic variant.

Two of the three IPAH patients, four HPAH patients and one HPAH family member were sequenced with a PAH-specific panel for 12 PAH genes and 17 candidate genes [20]. This identified a novel homozygous pathogenic *EIF2AK4* variant c.641delA p.(Lys214Argfs*21) in one family with two affected sisters clinically characterized as HPAH, albeit pulmonary veno-occlusive disease could not be excluded retrospectively. Further variants in the study cohort were reported previously (*BMPR2* c.224C>T p.Gln82*; *BMPR2* c.1453G>A p.Asp485Asn; *BMPR2* c.2695C>T p.Arg899*; *BMPR2* exon 2–3 deletion; *ENG* c.1633G>A p.Gly545Ser) [2,11,20].

## 4. Discussion

This study identified four novel and five further variants in the *BMPR2* promoter of I/HPAH patients. The transcriptional impact of these variants revealed a reduced transcriptional activity for seven out of nine variants (78%).

### 4.1. Impact of Promoter Variants on Transcription Factor Binding Sites

The variant c.-910C>T was identified for the first time in this study. This patient harbored not only the c.-910C>T but additionally the c.-933_-928dupGGCGGC variant in the *BMPR2* promoter region. These two variants were both shown to decrease transcriptional activity of *BMPR2*. The c.-910C>T variant was predicted to create a new binding site for the transcription factor Gli-similar 3 (*GLIS3*). Glis3 is a Krüppel-like transcription factor as a member of Gli and Zinc finger families which shows a tissue specific expression mainly in the endocrinal system such as the thyroid gland and in low levels also in the lung [22]. So far, no study reported the relation of Glis3 and the *BMPR2* gene or any PAH pathway related gene. However, the new PAH gene Krüppel-like transcription factor 2 (*KLF2*) could indicate a possible relation to this Zinc finger family member and PAH pathogenesis [23,24]. The promoter variant c.-910C>T did not cosegregate with PAH in the family but with a hypertensive PASP during exercise. In contrast, the second variant of the index patient in Family 1 (c.-933_-928dupGGCGGC) showed no cosegregation with an abnormal PASP response in family members and was less likely to have contributed to PAH manifestation.

The variant c.-1141C>T of *BMPR2* promoter was also newly identified in a HPAH patient and a significant reduction in the *BMPR2* promoter transcriptional activity expression was shown. In addition, in silico a loss of a pleomorphic adenoma gene 1 (*PLAG1*) transcription factor binding site was predicted. It was reported that *PLAG1* could act as an activator for a gene promoter by upregulating the insulin-like growth factor gene *IGF1* [25]. However, no study has so far elucidated the relation between the transcription factor *PLAG1* and the *BMPR2* gene. If it served as an activator of the *BMPR2* promoter, the loss of the *PLAG1* binding site predicted in this study would be consistent with the measured reduced transcriptional activity. However, the variant showed no cosegregation with the disease or an abnormal PASP response in the family, possibly only attributing a weak functional impact on the *BMPR2* gene and PAH manifestation. Reduced transcriptional activity without cosegregation in the family was also identified for the heterozygous variant c.-575A>T.

Moreover, this study identified three different types of GGC repeats in the c.-963_-928 upstream sequence of *BMPR2.* All of these variants led to a reduced transcriptional activity. The wild-type sequence at position c.-963_-928 upstream of transcription start site is made up of 12 tandem GGC repeats. Limsuwan and colleagues first discovered an abnormal number of GGC repeats within these sequences in congenital heart disease-associated PAH children [16]. The transcriptional assessment of the altered number of GGC tandem repeats in our functional analysis (11, 13 and 14 GGC repeats) in Family 1 and two IPAH patients were in line with another study showing a decrease in gene expression with a promoter containing 13 or 10 GGC repeats [21] in comparison to the 12-GGC repeat. A further study identified a GC>AT change within this repeat sequence cosegregating with the disease in an HPAH family [13]. The authors could establish that the variant led to a preferred cryptic translational start site, followed by a premature stop codon and nonsense mediated decay. This variant could not be identified within the GGC-repeat region in our study.

The “GGCG” sequence is a possible early growth response-1 (*EGR1*) transcription factor binding site. EGR1 binds to the promoter region of many target genes, thereby participating in various pathways such as those relevant for cardiovascular homeostasis [26]. This factor can serve as an activator or repressor and it can be activated by hypoxia in vitro [26]. Gaddipati et al. demonstrated an increase of EGR1 can lead to a decreased expression of *BMPR2* [27]. However, in another study, knockdown of the *EGR1* gene by short interfering RNA treatment resulted in a reduction of *BMPR2* promoter transcription activity [21]. Thus, the complex molecular mechanisms between the EGR1 and *BMPR2* promoter remain to be further investigated.

### 4.2. Contradictions to Previous Studies

The two variants c.-301G>A and c.-669G>A neither indicated a cosegregation with the disease nor showed any influence on gene transcription. This suggests that both variants may be benign. This is supported by the presence of both alleles in non-PAH controls in the gnomAD database (c.-301G>A: 0.69% in 217 out of 31,336 individuals; c.-669G>A: 0.95% in 297 out of 31,142 individuals). The functional study results contradicted a previous study, which had shown a reduced transcriptional activity of the c.-669G>A variant [14]. The variant was present in only two index patients of three HPAH families in our study. In one family it co-occurred with a pathogenic *BMPR2* exon 2–3 deletion and in the second family with a VUS in the *ENG* gene. Therefore, both the pedigree and functional results suggest a benign nature of the variant, also contradicting our own previous assumptions, which suggested the c.-669G>A variant contributed to disease manifestation [11].

For c.-301G>A, the segregation data and functional analysis showed opposing results to Pousada and colleagues, who reported a decreased gene expression in plasmids harboring the c.-301G>A variant [15]. A predicted loss of an Msh Homeobox 2 (MSX2) transcription factor binding site may result in a reduced *BMPR2* expression. So far, no study reported any relation between the MSH2 and *BMPR2* expression. A previous study demonstrated in Msx2^−/−^ mice an upregulation of *BMPR2* in smooth muscle cells in peripheral arteries [28]. Hence, the contradictory results above require further clarification to determine the effects of the promoter variants on *BMPR2* expression levels.

In contrast to the former studies, this study used a longer 5′UTR of the *BMPR2* gene as promoter sequence (1520 bp) in order to incorporate all detected variants and be able to compare them with each other in the same experimental set-up. In previous studies shorter 5′UTR fragments of 300 bp containing the region c.-770_c.-471 [14] and 539 bp including the base pairs c.-539_c.-1 [15] were used. A 5′UTR length-dependent up- or down-regulation of *BMPR2* has previously been described due to the inclusion of additional transcriptional modulators [27] and could have led to the different results. The origin of the cell culture cells used in functional analyses could also have influenced the results. In this study, PASMCs were used, which are at the heart of vascular remodeling in PAH, while in one of the two other studies the kidney cell line COS-1 was analyzed [15]. Expression data from different organs furthermore suggest the *BMPR2* gene expression in the lungs to be around three times higher than in the kidneys [29]. Thus, the different length of the promoter region encompassing different stretches and transcription factor binding sites of the *BMPR2* promotor, the different cell types and organ-specific gene expression may explain the different expression levels of the same variants between published works and this study. In future studies, expression levels should be compared in constructs with similar length of the inserted sequences and the same cell lines to have clearly comparable results to resolve the discrepancies.

### 4.3. Limitations and Future Directions

In this study, we only assessed one defined length of the promoter region in one specific cell line. Analyzing the effect of the promotor variants on *BMPR2*-gene expression using sequence fragments of different lengths in various cell lines side by side could help to elucidate the interaction of the transcription regulation domains within the promoter. Moreover, two promoter variants were identified in the same patient (Figure 3) but their effect was only analyzed separately. It would be interesting to evaluate their joint effect in the same plasmid and experimental setting to find out whether they are additive, lead to an even greater effect or cancel each other out.

In addition, the DNA-methylation of the promoter region should be measured in future studies to obtain a full picture of transcriptional activity. Equally, the evaluation of the subcellular localization of the respective BMPRII proteins could provide further functional evidence and valuable information regarding genetic variants in the *BMPR2* promoter region in future studies.

## 5. Conclusions

New variants in the *BMPR2* promoter region were discovered and investigated in this study. A down-regulated gene expression facilitated by variants in the *BMPR2* promoter suggested a possible impact on disease manifestation. However, the majority of variants showed no cosegregation with the disease or an elevated PASP during exercise in families, despite a statistically significant transcriptional impact. Thus, the promoter variants cannot fully explain the causality between the clinical manifestation and genotypes. There is still a lack of frequency data for promoter variants in a large PAH dataset. Predicted transcriptional factor binding sites remain to be validated and the complex mechanisms of altering these sites and the influence on gene transcription require further detailed investigations to determine the consequences on *BMPR2* expression levels. Thus, further studies are needed to investigate the frequency of *BMPR2* promoter variants and their impact on penetrance and disease manifestation.

## Figures and Tables

**Table 1:** Clinical characteristics of the index patients from the seven HPAH families at initial diagnosis.

Characteristic *	Mean ± SD	Min	Max
Women [%]	43		
Age at diagnosis [years]	34.0 ± 14.2	13	56
Heart rate [min^−1^]	88.6 ± 12.2	68	99
Oxygen saturation [%]	94.0 ± 5.0	87	98
Mean pulmonary artery pressure [mmHg]	60.3 ± 9.4	46	70
Pulmonary arterial wedge pressure [mmHg]	5.0 ± 1.6	3	7
Pulmonary vascular resistance [Wood Units]	20.2 ± 5.7	13	26
Cardiac index [ml/min/m^2^]	2.1 ± 0.9	1.3	3.6

* Not all measurements were obtained from each patient. SD: standard deviation.

**Table 2:** Variants identified in the 5′UTR of the *BMPR2* in I/HPAH patients.

Nucleotide Change	Patients	Variant Carriers	GnomAD Frequency	rsID	Described in PAH Patients
c.-301G>A ^1^	2 HPAH families	2 indices 6 family members ^2^	0.69%	rs116154690	1 SSc-APAH [15]
c.-575A>T	1 HPAH family	1 index	0.04%	rs550462760	This study
c.-586dupT ^3^	1 IPAH patient	1 index	0.17%	rs572725320	This study
c.-669G>A	3 HPAH families	2 indices 9 family members	0.95%	rs115604088	[11,14]
c.-910C>T ^4^	1 HPAH family	1 index 5 family members	-	-	This study
c.-930_-928dupGGC (13 repeats)	1 IPAH patient	1 index	-	rs375624016	4 CHD-APAH and 10 controls; 1 HPAH [16,21]
c.-933_-928dupGGCGGC (14 repeats) ^4^	1 HPAH family	1 index 2 family members	-	-	1 CHD-APAH [16]
c.-930_-928delGGC (11 repeats)	1 IPAH patient	1 index	-	rs886055459	1 CHD-APAH [16]
c.-1141C>T ^1^	1 HPAH family	1 index 2 family members	-	-	This study

^1^ c.-301G>A and c.-1141C>T were identified in the same HPAH family but in two different index patients of the same family. ^2^ All family members with any *BMPR2* promoter variant apart from one c.-301G>A carrier had no manifest PAH. ^3^ Homozygous variant, all other variants were heterozygous. ^4^ c.-910C>T and c.-933_-928dupGGCGGC were identified in the same HPAH family and index patient. CHD-APAH: congenital heart disease-associated pulmonary arterial hypertension; GnomAD: genome aggregation database; HPAH: heritable pulmonary arterial hypertension; IPAH: idiopathic pulmonary arterial hypertension; rsID: variant identifier; SSc-APAH: systemic sclerosis associated pulmonary arterial hypertension.

**Table 3:** *BMPR2* promoter and further gene variants in I/HPAH patients.

Family^−/−^ Index	Other Pathogenic Variants/VUS	*BMPR2* Promoter Variants	Gene Expression Compared to Wild-Type	Variant in FM with ↑ Exercise PASP/all FM with ↑ Exercise PASP	Variant in FM with Normal Exercise PASP/All FM with Normal Exercise PASP
Family 1	none	c.-910C>T	0.73 x	4/4	1/2
		c.-933_-928 dupGGCGGC	0.70 x	1/4	1/2
Family 2	*EIF2AK4* c.641delA p.(Lys214Argfs*21)	c.-1141C>T	0.79 x	in index patient	NA
		c.-301G>A	1.01 x	in 2nd PAH patient	NA
Family 3	none	c.-575A>T	0.60 x	NA	not in 2nd PAH patient
Family 4	none	c.-301G>A	1.01 x	1/2	1/1
Family 5 ^1^	*BMPR2* c.244C>T p.(Gln82*)	c.-669G>A	0.99 x	3/6	0/2
Family 6	*BMPR2* exon 2–3 deletion	c.-669G>A	0.99 x	4/6	0/2
Family 7	*ENG* c.1633G>A p.(Gly545Ser)	c.-669G>A	0.99 x	1/1	2/5
IPAH 1	none	c.-586dupT	0.80 x	NA	NA
IPAH 2	*BMPR2* c.1453G>A p.(Asp485Asn)	c.-930_-928delGGC	0.83 x	NA	NA
IPAH 3	*BMPR2* c.2695C>T p.(Arg899*)	c.-930_-928dupGGC	0.82 x	NA	NA

^1^ in Family 5, *BMPR2* c.-669G>A was identified in healthy family members but not in the index patient. In other families, variants listed were identified in the index patients. Exercise PASP was measured in 39 of 53 sequenced family members.↑: hypertensive; FM: family member; NA: no PASP measured in family members during exercise; PASP: pulmonary arterial systolic pressure; VUS: variants of uncertain significance. Reference sequence IDs: *BMPR2*: NM_001204, *EIF2AK4*: NM_001013703, *ENG*: NM_001114753.
